# Psychometric properties of the short Indonesian version of the Academic Stress Scale and descriptive analysis of academic-related stress among adolescent students

**DOI:** 10.3389/fpsyt.2026.1797437

**Published:** 2026-05-11

**Authors:** Tjhin Wiguna, Kusuma Minayati, Hans Christian, Adilla Hastika Fasha, Edo Sebastian Jaya, Fransiska Kaligis, Anthony Paul Sison Guerrero

**Affiliations:** 1 Child and Adolescent Psychiatry Division, Department of Psychiatry, Faculty of Medicine, Universitas Indonesia – Dr. Cipto Mangunkusomo General Hospital, Jakarta, Indonesia; 2 Psychiatry Subspecialty Residency Training, Department of Psychiatry, Faculty of Medicine, Universitas Indonesia – Dr. Cipto Mangunkusomo General Hospital, Jakarta, Indonesia; 3 Faculty of Psychology, Universitas Indonesia, Depok, West Java, Indonesia; 4 Department of Psychiatry, University of Hawaii, John A. Burns School of Medicine, Honolulu, HI, United States

**Keywords:** Academic-related stress, adolescent, Indonesian, reliability, stress scale, students, validation

## Abstract

**Purpose:**

The Academic Stress Scale (ASS) is widely used for screening academic-related stress among adolescent students; however, its 38 items may be too lengthy for practical application in school settings. Thus, this study aimed to evaluate the psychometric properties of the short Indonesian version of the ASS (SIVASS) and to identify the most prevalent academic-related stress among adolescent students.

**Method:**

This was a cross-sectional study that consisted of two steps. The first step included translation, back-translation, cultural adaptation, content validation, and item eligibility analysis to shorten the Indonesian version of the ASS. Content validity was assessed by five experts using the item-level content validity index (I-CVI), scale-level content validity index (S-CVI), and kappa statistics. The second step was conducted to evaluate the construct validation and reliability analysis of the SIVASS using exploratory factor analysis (EFA) and confirmatory factor analysis (CFA) and to identify the internal consistency. A total of 464 students from three schools in Jakarta were randomly divided into two subgroups (n = 232 each) for EFA and CFA. EFA and reliability analysis were conducted using SPSS version 29, and CFA was performed using Lavaan in R.

**Results:**

Based on the first step results, 18 items of the Indonesian version of the ASS were eligible for further construct analysis. However, EFA results only showed 16 items that were valid for the SIVASS and were categorized into four domains—parent-related, internal-related, friend-related, and teacher-related pressures—with all factor loadings exceeding 0.4. CFA indicated a good model fit (χ^2^ = 164.551, df = 100, p < 0.001). Convergent validity was acceptable [average variance extracted (AVE) = 0.73. Reliability analysis demonstrated a high internal consistency (Cronbach’s α = 0.91) and a strong agreement (interclass correlation coefficient = 0.899). “I worry about the possibility of disappointing my parents if I do not do well in school” caused the highest proportion of academic-related stress among current adolescents.

**Conclusion:**

The SIVASS is a valid and reliable 16-item screening questionnaire for assessing academic-related stress among Indonesian adolescents in diverse educational settings.

## 1 Introduction

Adolescence is a challenging period, especially for students. The transition from primary to secondary school may trigger several stressors, including academic demands, peer pressure, and teacher authority, in addition to the need to cope with expanding digital technologies, social media, and climate change (1, 2). Moreover, adolescence may increase stress due to physical, cognitive, emotional, and social changes associated with the maturation of the prefrontal cortex, which is responsible for judgment and self-regulation, as well as the rapid development of brain regions involved in sensation seeking and emotional processing (3). At the same time, adolescents are expected to achieve various developmental milestones, such as academic competence, healthy interpersonal relationships, and effective coping strategies for stress (4, 5). Hence, late primary and secondary school students experience a distinct life stage characterized by both increased challenges and opportunities.

Academic stress is defined as the pressure to achieve or perform in school during learning activities, examinations, or group discussions (6–8). The Organisation for Economic Co-operation and Development conducted a study involving 540,000 adolescent students, of whom 66% and 59% reported experiencing stress related to poor grades and worrying about tests even when well prepared, respectively (9). Furthermore, some students reported feeling uneasy while studying at school, and female students experienced higher levels of anxiety related to schoolwork compared to male students (9). The study also identified education and academic performance as significant sources of stress. A recent study further reported that students experience multiple sources of academic-related stress, including difficulties in understanding material, excessive workload, challenging examinations, problematic peer interactions, and frustration with school tasks and rules (10). Similar findings have been reported elsewhere, indicating that academic-related stress negatively affects quality of life and well-being (11). Moreover, undetected academic-related stress may impair conflict resolution skills and increase the risk of mental health problems, such as anxiety, depression, sleep disturbances, and substance use, among adolescents (11). Therefore, it is important to comprehensively understand academic-related stress, particularly in Asian countries that place strong emphasis on educational achievement, such as China, Singapore, South Korea, and Hong Kong (12). Similar patterns may also be observed among students in urban settings.

High expectations in academic settings may lead students to study more or adopt less effective learning strategies, contributing to academic-related stress (6, 13, 14). Sangma et al. (5) noted that such expectations often originate from parents, while adolescents may internalize these demands and experience additional pressure from teachers (4, 5). Late primary and secondary school students may be particularly affected, as they prepare for entry into desired schools, facing pressure driven by fear of failure, peer competition, and the need to meet parental and teacher expectations. Cassidy and Boulos (2023) further highlighted the association between academic expectations and students’ well-being, showing that heightened expectations may negatively impact psychological outcomes. For instance, over-involved and controlling parenting, often driven by parental ego fulfilment, may undermine students’ autonomy and well-being (15). Although not explicitly grounded in social cognitive theory, these findings align with Bandura’s framework, which suggests that environmental pressures shape students’ self-efficacy and influence their emotional and behavioral responses to academic demands (16).

The Academic Stress Scale (ASS), developed by West et al. (17), measures perceived academic situations or conditions that trigger anxiety or stress responses in students (18). This scale is unique in that it captures students’ perceptions of external pressures from teachers, parents, and peer groups rather than their self-perceptions. The ASS comprises 38 items categorized into four domains: fear of failure, peer pressure, importance of school, and parent-related pressure. Its reliability has been reported as relatively high (0.78). In 1990, Gozali (19) validated this questionnaire in the Indonesian language; however, the study was conducted in Palembang, South Sumatra Province, Indonesia. The results were categorized into two domains (internal and external pressures), which differ from the original structure proposed by West et al. Based on Gozali’s findings, the Indonesian version of the ASS demonstrated a solid theoretical foundation as a screening tool for academic-related stress (17, 20). However, a high correlation between the domains was observed, which may indicate overlap among items measuring similar constructs (20).

Nevertheless, the items of the ASS remain relevant to contemporary school contexts, particularly for identifying students’ perceptions of academic stressors. A key limitation is that the ASS comprises 38 items, which may be too lengthy and time-consuming for students to complete as a self-report questionnaire. Previous research and worldwide consensus suggest that 15–20 items may be sufficient for such measures (21). Therefore, shorter questionnaires may be more practical, efficient, and easier for students to complete. In studies involving a large battery of assessment tools, shorter instruments are advantageous, as they require less time to administer and score and are more likely to yield valid responses (22). Additionally, the Indonesian version of the ASS was translated between 1989 and 1990 and may now be outdated in terms of language use, including grammar, terminology, idioms, and expressions.

Thus, the present study aimed to evaluate the psychometric properties of the short Indonesian version of the ASS (SIVASS), including content validity, exploratory factor analysis, confirmatory factor analysis, and reliability analysis. Furthermore, this study sought to identify the most prevalent forms of academic-related stress experienced by adolescents today.

## 2 Methods

### 2.1 Study design

This cross-sectional study examined the psychometric properties of the SIVASS and identified the main sources of academic-related stress among adolescent students. All study protocols were approved by the Ethics Committee of the Faculty of Medicine, Universitas Indonesia–Dr. Cipto Mangunkusumo General Hospital, Jakarta, Indonesia (Reference No. KET-178/UN2.F1/ETIK/PPM.00.02/2024), in accordance with the ethical principles of the Declaration of Helsinki.

### 2.2 Academic stress scale

The ASS was a structured self-report questionnaire (17) that measured academic stress among adolescents. Originally designed as a Likert-type instrument, the ASS aimed to quantify the various sources of academic-related stress, such as peer pressure, parental expectations, personal concerns regarding academic performance, and fear of failure. This scale was validated via factor analysis and yielded four primary dimensions that encompass the perceived pressures that students experience academically. It comprises items rated on a 5-point Likert scale from 1 (strongly disagree) to 5 (strongly agree), which allows researchers to derive scores that indicate both total academic stress levels and stress related to specific domains.

The ASS has been validated in several countries and has highlighted its psychometric robustness across diverse cultural contexts, such as in the United States and England. Validation analyses, such as Coney and West (23), and cross-cultural studies (17) revealed that the ASS has high internal consistency (Cronbach’s alpha coefficients approximately 0.78), with sufficient reliability for measuring academic stress factors in adolescent populations (17, 23). The ASS is widely used in educational psychology, underscoring its versatility and ability to consistently assess academic-related stress, which contributes to its status as a reliable tool to understand the factors that affect students’ academic performance and psychological well-being (17, 19). In Indonesia, the scale was further validated through confirmatory factor analyses in 1990 and consisted of 38 items (19). However, there is no information regarding the content validity of the Indonesian ASS. Meanwhile, the usage is assumed to be limited in Indonesia due to the high number of items and longer time to fulfil. Thus, the shortened ASS is thought to be essential to increase its usability in schools.

### 2.3 Procedure and participants: first and second steps

Based on Marsh et al. (24), a good candidate questionnaire designed for shortening must have a sound theoretical foundation and significant goodness-of-fit constructs. The ASS fulfils these two requirements.

**Figure 1:**
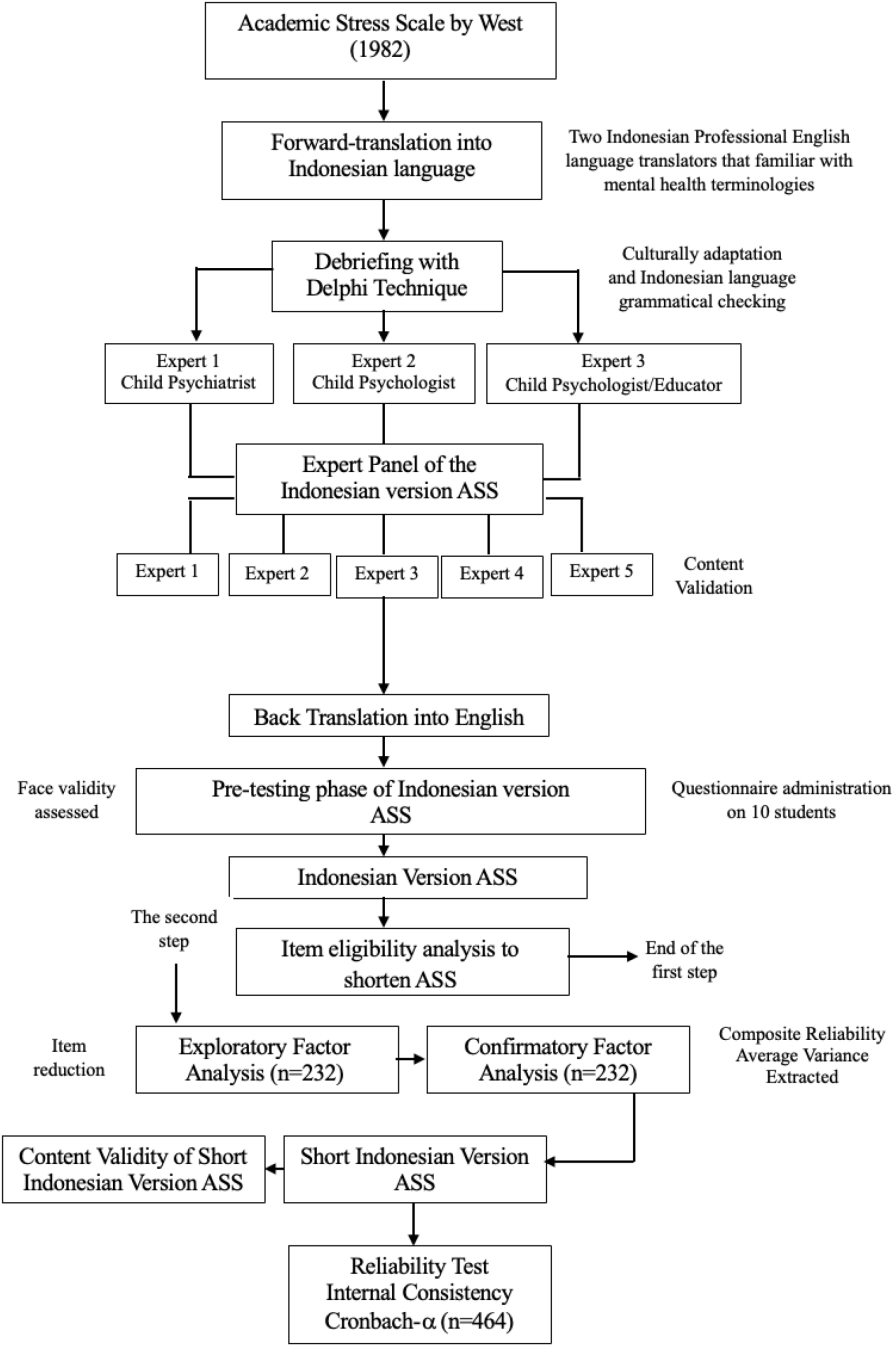
Research pathway for evaluating the psychometric properties of SIVASS. SIVASS, short Indonesian version of the academic stress scale.

The first step consisted of translation and adaptation of the ASS into the Indonesian language, a content validation, and an item eligibility study to shorten the ASS. The second step identified the SIVASS construct validity and reliability. This included exploratory factor analysis (EFA), confirmatory factor analysis (CFA), and, finally, reliability analysis. **Figure 1** illustrates the first and second research pathways.

The first step of this study started with the translation and adaptation of the ASS into an Indonesian context. A cognitive debriefing involved two child psychologists and one child and adolescent psychiatrist who assessed the relevance and cultural adaptability of the ASS for Indonesian usage. Subsequently, five experts (three child and adolescent psychiatrists, one psychologist, and one secondary high school teacher) conducted the content validation of the Indonesian version of the ASS. Pilot testing was conducted with 10 students, which enabled refinement based on direct feedback. Back-translation was performed to ensure the alignment with the original content. Finally, the first step ended with item eligibility analysis to shorten the Indonesian version of the ASS.

The second step aimed to identify the construct validity and reliability of the SIVASS. This began with item eligibility analysis. EFA, CFA, and reliability analysis involved 464 students (grades 5 and 6 of primary and secondary high school students) from Jakarta, Indonesia. Participants were randomly divided into two equal groups for EFA (n = 232) and CFA (n = 232). In total, three schools participated: a government primary school, a government secondary high school, and a private secondary high school. Before the study began, parents, school headmasters, and teachers were informed of the study’s aims, method, and process. After the headmaster of each school approved, students and parents who were willing to participate were selected. The inclusion criteria for EFA and CFA were students 1) from grades 5 and 6 of the primary school and secondary high school, 2) who had a normal intelligence quotient based on Raven’s Standard Progressive Matrices, 3) who were willing to participate after they signed the informed assent form, and 4) for whom informed consent was obtained from their parents, which was witnessed by the teachers.

### 2.4 Statistical analysis

#### 2.4.1 Content validation

Five experts assessed the content validity of the Indonesian version of the ASS. The panel comprised three child and adolescent psychiatrists, one psychologist, and one secondary high school teacher with more than 10 years of experience in handling academic issues among children in Indonesia. Content validity evaluation employed the Delphi technique, in which each expert independently rated each item on a 4-point Likert scale that ranged from 1 (strongly disagree) to 4 (strongly agree). Quantification of content validity involved the use of the content validity index (CVI) and the kappa statistic. The CVI was computed for individual items (I-CVI) as well as for the entire scale (S-CVI/Ave). The I-CVI for each item was determined by dividing the number of experts rating the item as 3 (agree) or 4 (strongly agree) by the total number of experts. The cut-off for the I-CVI was established as 0.79. To confirm the content validity of the overall scale, the S-CVI was calculated by averaging the I-CVI values. The kappa statistic, which complemented the CVI, served as a consensus index of inter-rater agreement, which ensured that agreement among the experts was not due to chance. Values >0.74, 0.6–0.74, and 0.4–0.59 were categorized as excellent, good, and fair, respectively. Items were eliminated if the kappa score was <0.4.

To shorten the Indonesian ASS, the study set two requirements based on Hair et al. (25) and Kim (26): 1) delete any datasets that contained >5% missing values. However, no datasets had any missing values. 2) Eliminate items that exhibit unacceptable item–total correlation (*r_it_*  <  0.50), skewness (>|2|), or kurtosis (>|4|).

#### 2.4.2 Exploratory factor analysis

This study used EFA to identify significant items in a certain factor structure of the SIVASS. EFA was conducted using SPSS version 29. Bartlett’s test of sphericity was employed to confirm that the correlation matrix was not random. Furthermore, the Kaiser–Meyer–Olkin (KMO) statistic was required to exceed a minimum threshold of 0.50. Communality assessments for each item were also conducted to identify any items to be excluded; items with communalities <0.5 were excluded. Factor extraction was performed using the principal component method. The optimal number of factors was determined by identifying an “elbow” in a scree plot, namely, where the curve’s slope clearly decreases. Item allocation was performed via varimax rotation.

#### 2.4.3 Confirmatory factor analysis

**Figure 2:**
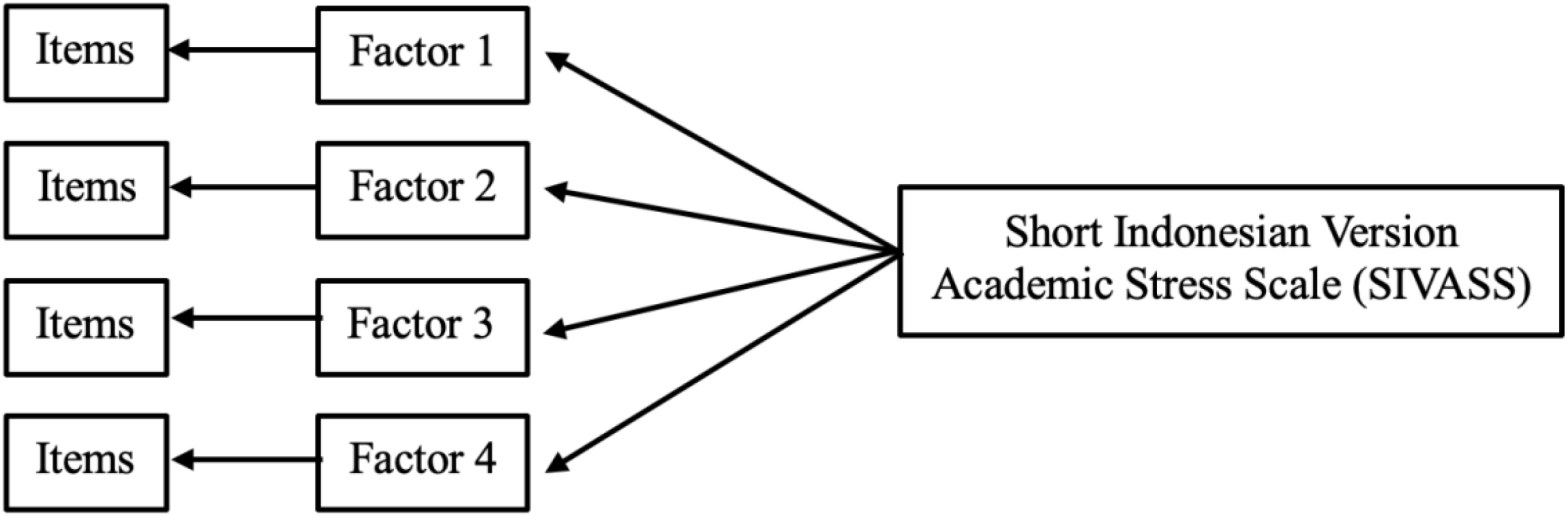
Hypothesized construct validity model of SIVASS. SIVASS, short Indonesian version of the academic stress scale.

To evaluate the construct validity of the EFA model, we conducted CFA using the maximum likelihood estimation method. CFA was an appropriate statistical technique, as it allowed us to assess a predefined factor structure and evaluate how well the observed variables represented the underlying latent constructs. We performed CFA using Lavaan in R. We hypothesized a four-factor model based on West (16). We assumed factor loadings of the observed variables on their respective latent factors to be non-zero, which reflected the relationship between the constructs and indicators. Additionally, we allowed the residual variances of the indicators to be correlated when theoretically justified. **Figure 2** illustrates the hypothesized model. We constrained the loadings of the first indicator for each latent factor to 1 to serve as a reference, as is typical in a CFA. We employed the maximum likelihood (ML) method to estimate the parameters of the CFA model. We evaluated model fit using a combination of several goodness-of-fit indices:

- chi-squared test (χ^2^ ≥ 3.84, p < 0.05): to assess the overall fit of the model.
- Comparative fit index (CFI): values >0.90 indicated good fit.
- Tucker–Lewis index (TLI): values >0.90 suggested an acceptable fit.
- Root mean square error of approximation (RMSEA): values <0.08 suggested a good fit.
- Standardized root mean square residual (SRMR): values <0.08 indicated a good fit.

Factor internal consistency was assessed using composite reliability (CR) and average variance extracted (AVE) for convergent validity. The CR of the SIVASS was set above the recommended threshold of 0.70, which indicates good reliability. The AVE was set as ≥0.50, which suggests adequate convergent validity.

#### 2.4.4 Reliability analysis

Reliability analysis was conducted using SPSS version 29 and included the identification of internal consistency (Cronbach’s α) and intraclass correlation coefficients (ICCs). Test–retest reliability was examined in a randomly selected subsample of 30 students drawn from the total sample (n = 464). The same participants completed the SIVASS again 2 weeks after the initial administration, and paired scores were analyzed to assess temporal stability using the ICC.

## 3 Results

The first step comprised translation, a debriefing session, pre-testing with students, content validity, and item eligibility analysis to shorten the Indonesian version of the ASS. During the debriefing session, the context of the Indonesian version of the ASS was considered culturally appropriate. Therefore, the forward translation into the Indonesian language was accepted with minor grammatical revision. During the pre-testing period, the 10 students from the fifth grade and secondary high school level had no difficulties in understanding each item. Moreover, students mentioned that no items raised any uncomfortable feelings, but 38 items of the ASS were quite challenging to complete.

Content validity was conducted by three child and adolescent psychiatrists, one psychologist, and one secondary high school teacher. Experts evaluated the 38 items of the Indonesian version of the ASS; 29 items had a good I-CVI (>0.78), five had low I-CVI values (<0.78) with a fair kappa score (>0.40), and four had an I-CVI <0.78 with an unacceptable kappa score (<0.4). Five items were included (items 21, 26, 28, 29, and 35), and four items were excluded (items 14, 23, 37, and 38; **Supplementary Material**). The S-CVI of the Indonesian version of the ASS with 34 items was 0.84.

Hence, 34 items of the Indonesian version of the ASS were included in the eligibility analysis to shorten the Indonesian version of the ASS. The eligibility analysis of the 34 items of the Indonesian version of the ASS was based on the studies of Hair et al. (24) and Kim (25). Results revealed that all items exhibited acceptable skewness and kurtosis values. However, 16 items had item–total correlation values <0.5 and were excluded. Hence, only 18 items were included in EFA (**Table 1**).

**Table 1:** Items’ eligibility results.

Item	Item–total correlation	Skewness	Kurtosis
5. I frequently worry about the restrictions my parents will put on me if I don’t keep my grades up. *	0.573	−0.14	−1.08
8. I feel upset when my classmates find out about a low grade I have received in school.	0.599	−0.19	−0.96
9. It is very frustrating for me when I can’t seem to learn the things I’m supposed to for school.	0.562	−0.37	−0.74
11. I often worry about what my parents will say when they see the grades I receive on my report card.	0.615	−0.56	−0.52
12. I hate the thought of having to tell my parents about a test I haven’t done well on in school.	0.571	0.16	−0.94
14. It upsets me when I can’t understand the assignments my teacher gives at school.	0.594	−0.36	−0.75
15. I usually worry about what my friends and classmates think of me when the teacher calls on me and I don’t know the answer. *	0.526	−0.4	−0.59
17. It would bother me if my parents were to ask to see a test that I had done poorly on.	0.537	0.2	−0.96
18. I would worry about what my parents would do to me if they saw a low grade I received at school.	0.642	−0.11	−0.97
19. It embarrasses me when the kids at school make fun of me because I can’t answer a question in class.	0.586	−0.04	−1.09
21. It bothers me when my friends ask me about a test I have done poorly on.	0.556	−0.03	−0.85
23. It would frustrate me if my parents told me that I should be able to make better grades at school.	0.575	0.18	−0.99
25. It would upset me if my teacher had to talk to me about a low grade I had received in school.	0.515	0.1	−0.99
27. It bothers me quite a bit when I don’t do well in school because I’m afraid that my friends and classmates will think I’m stupid.	0.524	0.05	−1
29. I often worry about the possibility of not doing well enough in school to get into college.	0.572	−0.6	−0.27
30. I worry about the possibility of disappointing my parents if I don’t do well in school.	0.600	−0.87	0.37
31. I would worry about what my parents would do to me if my teacher had to notify them about my work at school.	0.593	−0.14	−0.82
32. It would disturb me if my teacher said I wasn’t trying in class because I didn’t do as well as the school thought I should do.	0.530	0	−0.87

All items have total correlation (*r_it_*  > 0.50), skewness (<|2|), and kurtosis (<|4|); included in EFA.

EFA, exploratory factor analysis; SIVASS, short Indonesian version of the Academic Stress Scale.

*Items 5 and 15 were subsequently removed during EFA due to low communalities, resulting in the final 16-item SIVASS.

### 3.1 Exploratory factor analysis

EFA included 232 participants, with a mean age of 13.42 years (95% CI: 13.1–13.8). Among them, 45 (19.40%, 95% CI: 14.8–24.9) and 187 (80.60%, 95% CI: 75.0–85.2) were elementary and junior high school students, respectively.

EFA results revealed that Bartlett’s test of sphericity had a χ^2^ value of 3,048.932 (p < 0.001), which indicated that the correlation matrix was not random. Additionally, the KMO value was 0.906, which exceeded the minimum acceptable threshold. Among the 18 items analyzed, two items (items 5 and 15) exhibited communality values <0.50 and were excluded. Subsequent factor analysis yielded a χ^2^ value of 2,574.713 (p < 0.001) and a KMO value of 0.901, which indicated a robust structure for further analysis. All items had communality values >0.50, which confirmed their adequacy for inclusion. Based on the scree plot (**Supplementary Material**), the optimal number of factors was determined as four. **Table 2** presents a detailed allocation of each item across these factors.

**Table 2:** EFA result*.

Item	Parent-related pressure	Internal-related pressure	Friend-related pressure	Teacher-related pressure
I hate the thought of having to tell my parents about a test I have not done well on in school (Item 12)	0.786			
I would worry about what my parents would do to me if they saw a low grade I received at school (Item 18)	0.744			
It would bother me if my parents were to ask to see a test that I had done poorly on in school (Item 17)	0.691			
It would frustrate me if my parents told me that I should be able to make better grades at school (Item 23)	0.636			
I would worry about what my parents would do to me if my teacher had to notify them about my work at school (Item 31)	0.605			
I often worry about what my parents will say when they see the grades I receive on my report card (Item 11)	0.553			
I often worry about the possibility of not doing well enough in school to get into college (Item 29)		0.73		
It is very frustrating for me when I cannot seem to learn the things I am supposed to for school (Item 9)		0.71		
It upsets me when I cannot understand the assignments my teacher gives at school (Item 14)		0.682		
I worry about the possibility of disappointing my parents if I do not do well in school (Item 30)		0.659		
It embarrasses me when the kids at school make fun of me because I cannot answer a question in class (Item 19)			0.769	
It bothers me when my friends ask me about a test I have done poorly on (Item 21)			0.715	
It bothers me quite a bit when I do not do well in school because I am afraid that my friends and classmates will think I am stupid (Item 27)			0.654	
I feel upset when my classmates find out about a low grade I have received in school (Item 8)			0.633	
It would disturb me if my teacher said I was not trying in class because I did not do as well as the school thought I should do (Item 32)				0.805
It would upset me if my teacher had to talk to me about a low grade I had received in school (Item 25)				0.678

EFA, exploratory factor analysis.

*Extraction method: principal component analysis; rotation method: varimax.

Following EFA, 16 items of the SIVASS demonstrated the strongest eigenvalue support for a four-domain structure (**Table 2**), namely, parent-related pressure, internal-related pressure, friend-related pressure, and teacher-related pressure. Each item within these domains demonstrated factor loadings that exceeded 0.4, which indicated strong internal consistency across the domains. This structure was subsequently confirmed via CFA, which further validated the model’s robustness in capturing SIVASS factors.

### 3.2 Confirmatory factor analysis

CFA was performed using a second independent subsample consisting of 232 students, with a mean age of 13.45 years (95% CI: 13.3–13.6). Among them, 34 (15.25%, 95% CI: 11.1–20.6) and 189 (84.75%, 95% CI: 79.4–88.9) were elementary and junior high school students, respectively.

**Figure 3:**
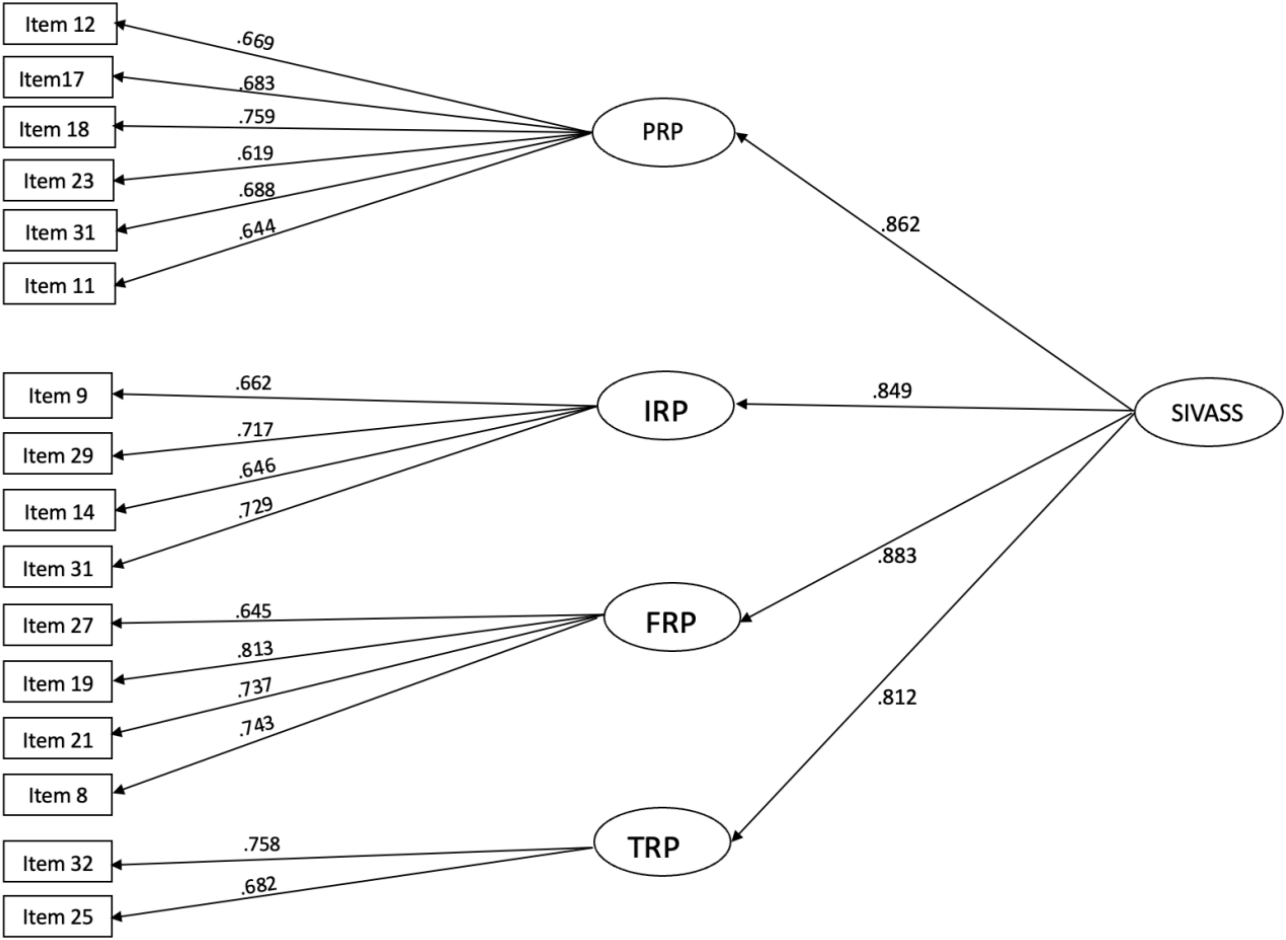
Confirmatory factor analysis of SIVASS. χ^2^ = 164.551, df = 100, p < 0.001; CFI = 0.957; TLI = 0.948; RMSEA = 0.053; SRMR = 0.045; CR = 0.91; AVE = 0.725. SIVASS, short Indonesian version of the Academic Stress Scale; PRP, parent-related pressure; IRP, internal-related pressure; FRP, friend-related pressure; TRP, teacher-related pressure; CFI, comparative fit index; TLI, Tucker–Lewis index; RMSEA, root mean square error of approximation; SRMR, standardized root mean square residual; CR, composite reliability; AVE, average variance extracted.

CFA results indicated that the hypothesized model and data fit well for the SIVASS (**Figure 3**). The chi-square test yielded a statistically significant value (χ^2^ = 164.551, df = 100, p < 0.001), which was expected, given the test’s sensitivity to large sample sizes. Additional fit indices further supported the model’s adequacy: the CFI was 0.957, and the TLI was 0.948, both of which exceeded the threshold of 0.95, which indicated good fit. The RMSEA was 0.053, with a 90% confidence interval that ranged from 0.038 to 0.067, and the SRMR was 0.045, both of which were within acceptable limits. The four-domain structure exhibited strong support, with all standardized factor loadings exceeding 0.6, which demonstrated the robustness of the measurement model. Convergent validity was sufficient (AVE = 0.73). **Figure 3** illustrates the detailed allocation of items across the four factors.

Finally, based on the similar judgements of the experts, the content validity of the SIVASS was high (S-CVI = 0.90). Moreover, internal consistency (Cronbach’s α) of the SIVASS was 0.91, and the interclass correlation was 0.899 (95% confidence interval = 0.855–0.912).

### 3.3 Characteristics of academic-related stress among adolescent students

**Figure 4:**
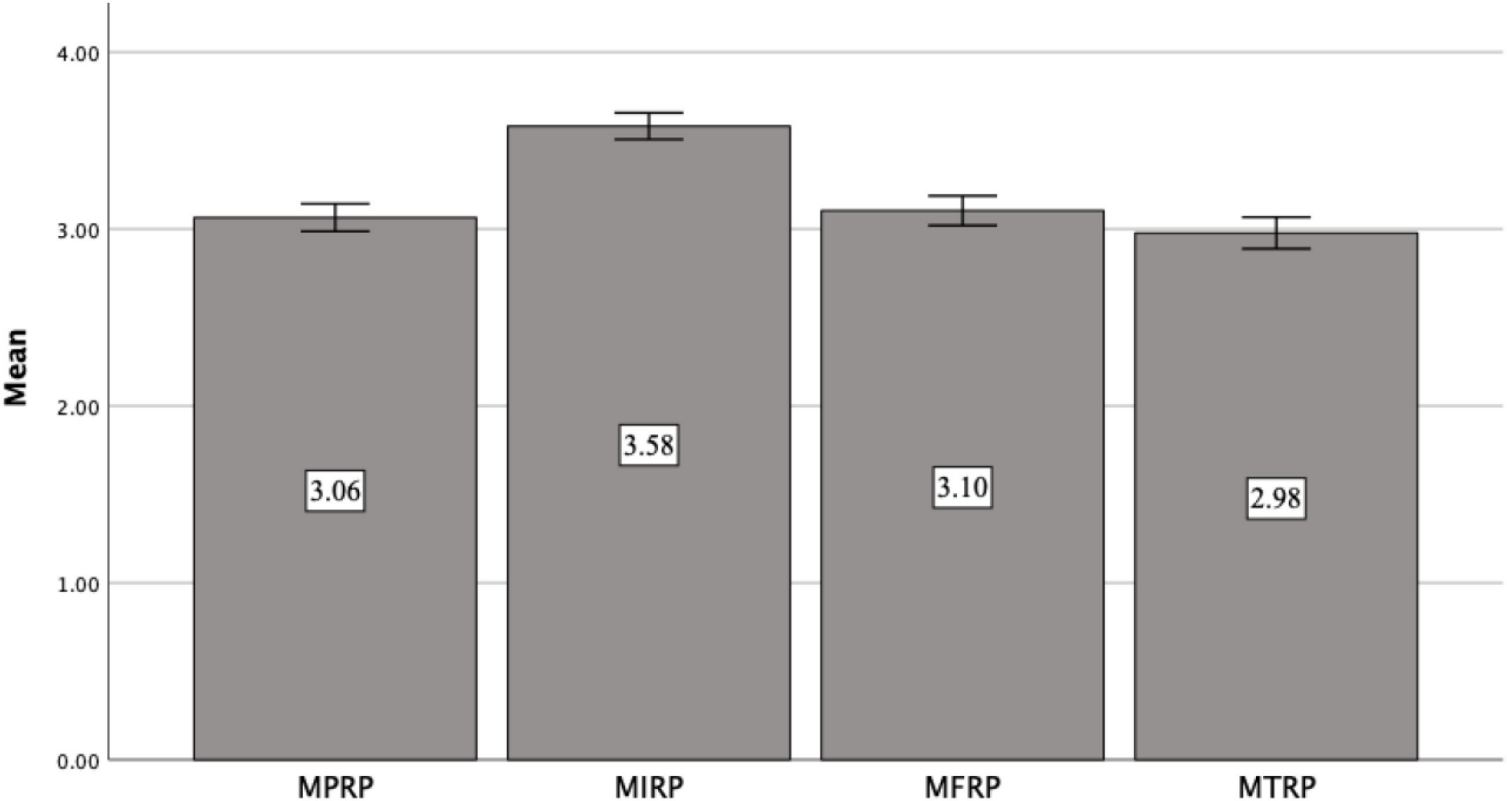
Domain-level mean scores of SIVASS. SIVASS, short Indonesian version of the academic stress scale.

The mean score of the total SIVASS was 51.05 (95% CI: 49.49–52.61). **Figure 4** provides a visual representation of the domain mean scores. The *internal-related pressure* (IRP) domain demonstrated the highest mean score (3.58; 95% CI: 3.51–3.66). Both the *friend-related pressure* (FRP) and *parent-related pressure* (PRP) domains exhibited comparable mean scores, with values of 3.10 [95% CI: 3.02–3.19] and 3.06 [95% CI: 2.99–3.14], respectively. The *teacher-related pressure* (TRP) domain had the lowest mean score (2.98; 95% CI: 2.89–3.06).

**Figure 5:**
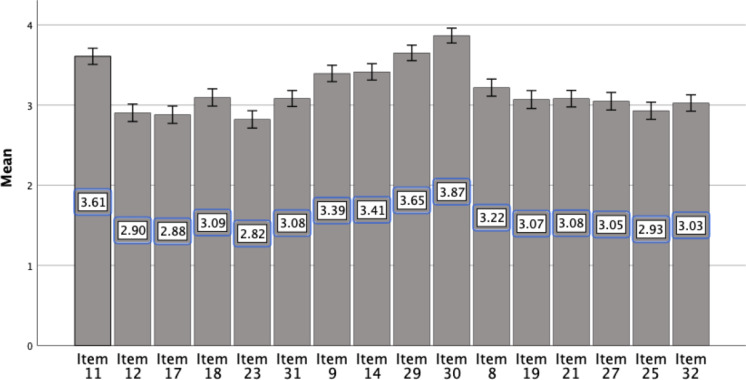
Item-level mean scores of SIVASS. SIVASS, short Indonesian version of the academic stress scale.

In addition to domain-level analysis, item-level mean scores were examined to identify the items with the highest mean scores. **Figure 5** presents the results of the item-level mean score analysis. Items from the SIVASS with the highest mean scores (SD) were item 30 (3.87; 95% CI: 3.76–3.96), item 29 (3.65; 95% CI: 3.55–3.74), and item 11 (3.61; 95% CI: 3.51–3.71). Conversely, the item with the lowest mean score was item 23 (2.82; 95% CI: 2.72–2.92).

The proportion of students reporting different types of academic-related stress was also assessed (**Table 3**). Academic-related stress was defined as students with Likert scale ratings of 4 (agree) and 5 (strongly agree) on the SIVASS. Consistent with the mean scores, item 30, “I worry about the possibility of disappointing my parents if I do not do well in school”, exhibited the highest proportion (73.27%; 95% CI: 67.67%–78.45%), followed by items 29, “I often worry about the possibility of not doing well enough in school to get into college” (62.93%; 95% CI: 56.90%–68.97%), and 11, “I often worry about what my parents will say when they see the grades I receive on my report card” (62.50%; 95% CI: 56.47%–68.53%). In contrast, item 12, “I hate the thought of having to tell my parents about a test I have not done well on in school”, had the lowest proportion (29.30%; 95% CI: 23.28%–35.33%) (**Table 3**).

**Table 3:** Proportion of different types of academic-related stress among adolescent students.

Domain	Item	Proportion (%)	95% CI (%)
Parent-related pressure	I often worry about what my parents will say when they see the grades I receive on my report card.	62.5	56.47–68.53
	I hate the thought of having to tell my parents about a test I have not done well on in school.	29.30	23.28–35.33
	It would bother me if my parents were to ask to see a test that I had done poorly on in school.	35.3	28.89–41.81
	I would worry about what my parents would do to me if they saw a low grade I received at school.	42.7	35.78–49.57
	It would frustrate me if my parents told me that I should be able to get better grades at school.	30.2	24.14–36.21
	I would worry about what my parents would do to me if my teacher had to notify them about my work at school.	36.6	30.60–43.10
Internal-related pressure	It is very frustrating for me when I cannot seem to learn the things I am supposed to for school.	50.0	43.10–56.90
	I often worry about the possibility of not doing well enough in school to get into college	62.9	56.90–68.97
	It upsets me when I cannot understand the assignments my teacher gives at school.	51.7	45.69–58.62
	I worry about the possibility of disappointing my parents if I do not do well in school.	73.3	67.67–78.45
Friend-related pressure	It embarrasses me when the kids at school make fun of me because I cannot answer a question in class.	40.5	34.05–46.98
	It bothers me when my friends ask me about a test I have done poorly on.	40.9	34.05–47.83
	It bothers me quite a bit when I do not do well in school because I am afraid that my friends and classmates will think I am stupid.	34.9	28.88–40.95
	I feel upset when my classmates find out about a low grade I have received in school.	46.6	39.66–53.02
Teacher-related pressure	It would upset me if my teacher had to talk to me about a low grade I had received in school.	36.6	30.18–43.09
	It would disturb me if my teacher said I was not trying in class because I did not do as well as the school thought I should do.	32.8	26.29–38.79

## 4 Discussion

This study found that the SIVASS consisted of 16 items and did not have any cultural issues based on discussion with experts during the debriefing session. The items reflect academic pressures commonly experienced by students across general contexts, such as “I would worry about what my parents would do to me if my teacher had to notify them about my work at school”, “I often worry about the possibility of not doing well enough in school to get into college”, and “It bothers me when my friends ask me about a test I have done poorly on”. Thus, no cultural discrepancies were identified across the 16 items, and the content validity was acceptable. Meanwhile, its internal consistency (Cronbach’s α) was 0.91, which indicated good reliability when administered repeatedly under the same conditions to the same individuals (27). This finding supports the dependability of the SIVASS in measuring academic-related stress.

Furthermore, the culturally adapted SIVASS demonstrates an appropriate construct for identifying academic-related stress among Indonesian adolescent students. The instrument comprises 16 items across four domains, consistent with the original version developed by West et al. (17). Based on EFA and CFA, the four domains are parent-related, internal-related, friend-related, and teacher-related pressures. As a multidimensional instrument, the SIVASS may offer stronger validity and reliability compared to similar tools, as supported by the analysis. Academic pressure is a complex phenomenon involving multiple factors, and academic-related stress can be understood as the result of interactions among academic demands, students’ coping capacities, and environmental resources (6). Therefore, a multidimensional approach provides a more comprehensive framework, allowing academic-related stress to be categorized into more manageable and interpretable components.

Thus, the SIVASS demonstrates superior construct validity. Gozali’s study (19) reported several items exhibited factor loadings below 0.4, indicating suboptimal representation of the intended domains. Consequently, adjustments to both the domain structures and item composition were required. In contrast, the present study showed a significant improvement in construct validity, with all items demonstrating adequate factor loadings, thereby confirming the robustness of the SIVASS. Moreover, the SIVASS comprises only 16 items and exhibits superior reliability, as evidenced by higher internal consistency indices compared to the ASS. These findings suggest that the SIVASS is a psychometrically sound and efficient instrument for assessing academic-related stress.

Furthermore, the SIVASS demonstrates strong validity and reliability compared to other measures of academic-related stress. Previous studies by Sun et al. (28), Levenstein et al. (29), Byrne et al. (30), and Mohamed Arip et al. (31) validated alternative stress scales, reporting comparable or slightly lower goodness-of-fit indices, thereby supporting the satisfactory validity of the SIVASS. However, these instruments have not been validated in the Indonesian language. An Indonesian validation study by Izzati et al. (32), based on Sarafino and Smith (33), developed a 24-item Academic Stress Scale covering biological, cognitive psychosocial, emotional psychosocial, and behavioral domains. Notably, this scale primarily measures stress responses (e.g., difficulty concentrating, forgetfulness, and negative thinking), whereas the SIVASS focuses on students’ perceptions of academic environments as psychosocial stressors. Thus, the two instruments reflect different conceptualizations of academic stress. Additionally, Stankovska et al. (34) developed the Student Academic Stress Test (SAST), which contains 70 items. In comparison, the SIVASS, with only 16 items, is more practical and may reduce respondent fatigue. Moreover, the SAST has not been validated in the Indonesian language.

In this study, the three most prevalent academic-related stressors were “I worry about the possibility of disappointing my parents if I do not do well in school”, which exhibited the highest proportion, followed by “I often worry about the possibility of not doing well enough in school to get into college” and “I often worry about what my parents will say when they see the grades I receive on my report card”. The results showed that internal- and parent-related pressures were the prominent stressors, representing major academic challenges among students. Previous studies have indicated that high school students experience a higher level of stress than younger students, particularly when facing high-stakes examinations at the end of their high school education (11, 35). This condition may be related to concern about achieving good results and making appropriate choices for college majors, as opportunities to enter reputable schools or universities are limited, and failure to do so may negatively affect future career prospects (15, 36–38). Consistent with the high mean scores for internal-related stress on the SIVASS, the present findings suggest that feelings of guilt, worry, and perceived incompetence remain prevalent among students. Previous studies have shown that internal-related academic stress is associated with conflicting thoughts, emotions, and self-imposed expectations that influence academic performance. Such conditions often lead to increased anxiety and depressive symptoms due to the persistent pressure to succeed while managing personal doubts (39–41). Moreover, similar to other Asian countries, Indonesia places strong emphasis on parental involvement in education. Many Asian cultures are characterized by a collectivist orientation, in which families hold a central position in shaping children’s educational experiences. Parents often strive to raise children who contribute positively to the family, leading to high expectations for academic achievement that benefit both the individual and the broader family or community (42, 43).

Moreover, Asian parents often experience considerable sociocultural pressure to ensure that their children achieve high academic performance, which is perceived as a reflection of parental competence and familial honor (42, 44). Within collectivistic cultures, particularly in many Asian societies, a child’s achievements are not merely individual accomplishments but are viewed as extensions of the family’s reputation. Consequently, academic success becomes a highly valued goal for both the child and the family unit. According to the transactional model of stress and coping proposed by Lazarus and Folkman (1984), stress arises when individuals appraise environmental demands as exceeding their coping resources (45). In this context, students may perceive parental expectations as a chronic source of pressure. Although poor academic performance can be stressful, it is often the anticipated parental response—such as disappointment or increased demands to study—that intensifies emotional strain.

In this study, the lowest proportion of academic-related stress was “I hate the thought of having to tell my parents about a test I have not done well in”. Although this may initially suggest that parental reactions are not a major source of academic stress, it may instead reflect a deeper coping adaptation. Based on the broader Indonesian cultural context, which is predominantly collectivist, maintaining family harmony and showing respect toward authority figures such as parents, older individuals, and teachers are highly valued. Consequently, adolescents may be less likely to openly express statements that could be perceived as disrespectful or upsetting. Instead, they may rely on emotion-focused coping strategies, such as avoidance or emotional suppression, to manage anticipated distress. This cultural tendency may explain the lower level of reported agreement with this item, underscoring the importance of interpreting these findings within their sociocultural context.

From a theoretical standpoint, this phenomenon can be interpreted using Bandura’s self-efficacy theory (1997), which posits that repeated negative feedback or lack of control in academic contexts can reduce students’ belief in their own abilities to influence outcomes (16). As a result, students may disengage emotionally from situations where they expect negative parental reactions, leading to the use of emotion-focused coping strategies, such as suppression, avoidance, or resignation (45). This form of coping may reduce acute distress in the short term but could contribute to long-term psychological difficulties, including low academic motivation or internalized shame. Taken together, these findings underscore the complex interplay between cultural expectations, cognitive appraisal, and coping strategies in shaping students’ experiences of academic stress. What may appear as a low-stress response in quantitative data may, in fact, conceal nuanced emotional processes rooted in both familial dynamics and culturally shaped coping styles.

This study had several strengths. First, it is a study to shorten the ASS, making it easier and more practical to apply in school settings. Moreover, the findings on academic-related stress may serve as a reference for future research and for the development of school-based mental health programs grounded in students’ perceptions. However, several limitations should be noted. The study included students from three schools, and the number of participants may be relatively limited. Nevertheless, the sample comprised students from both public and private schools in Jakarta, including primary school students (grades 5 and 6) and secondary school students (grades 1 to 3). This composition provides a reasonably heterogeneous sample for a validation study, particularly given that Jakarta, the capital city of Indonesia, is a highly multicultural city. Although the number of participants was relatively small, EFA and CFA demonstrated satisfactory goodness-of-fit indices, as well as acceptable CR and AVE values.

In conclusion, these psychometric evaluation of the SIVASS demonstrates that this 16-item questionnaire is a valid and reliable screening tool for identifying academic-related stress among Indonesian adolescent students. Given its sound psychometric properties, the SIVASS can be applied in primary, secondary, and high school settings to facilitate the early detection of academic-related stress. Consistent with previous studies, the current findings confirm that feelings of academic inadequacy and concerns about future outcomes remain significant sources of stress among adolescents. These findings underscore the importance of strengthening school-based mental health programs, particularly those that support the development of adaptive coping strategies tailored to diverse stressors. Such programs should also address the informational and emotional needs of parents and teachers. Furthermore, interventions should aim to enhance parental and teacher understanding of students’ academic capabilities, which may help reduce conflict and improve support for adolescents’ psychological well-being. Further study should include a larger and more heterogeneous sample, examine coping mechanisms related to academic stress, and evaluate targeted intervention, particularly through a longitudinal study design to better capture changes over time.

## Data Availability

The raw data supporting the conclusions of this article will be made available by the authors, without undue reservation.
