# Supplementary material for: Psychometric properties of the short Indonesian version of the Academic Stress Scale and descriptive analysis of academic-related stress among adolescent students
Source: Front Psychiatry. 2026 May 11;17:1797437. doi: 10.3389/fpsyt.2026.1797437 (PMC13200604; doi:10.3389/fpsyt.2026.1797437)
Supplement: Supplementary file 1 [file SupplementaryFile1.docx]

Supplementary File

Table 1. Indonesian version ASS Content Validation Results

| Item | Expert 1 | Expert 2 | Expert 3 | Expert4 | Expert 5 | I-CVI | Kappa | CVR |
| --- | --- | --- | --- | --- | --- | --- | --- | --- |
| 1 | 3 | 4 | 4 | 3 | 4 | 1.00 | 1.00 | 1.00 |
| 2 | 3 | 4 | 4 | 3 | 4 | 1.00 | 1.00 | 1.00 |
| 3 | 2 | 3 | 4 | 3 | 4 | 0.80 | 0.76 | 0.60 |
| 4 | 3 | 4 | 4 | 3 | 4 | 1.00 | 1.00 | 1.00 |
| 5 | 3 | 3 | 4 | 2 | 4 | 0.80 | 0.76 | 0.60 |
| 6 | 3 | 4 | 4 | 2 | 4 | 0.80 | 0.76 | 0.60 |
| 7 | 3 | 3 | 3 | 4 | 1 | 0.80 | 0.76 | 1.00 |
| 8 | 3 | 4 | 4 | 2 | 4 | 0.80 | 0.76 | 0.60 |
| 9 | 2 | 4 | 3 | 3 | 4 | 0.80 | 0.76 | 0.60 |
| 10 | 3 | 4 | 4 | 3 | 4 | 1.00 | 1.00 | 1.00 |
| 11 | 3 | 4 | 4 | 3 | 4 | 1.00 | 1.00 | 1.00 |
| 12 | 3 | 3 | 4 | 3 | 4 | 1.00 | 1.00 | 1.00 |
| 13 | 3 | 4 | 4 | 3 | 4 | 1.00 | 1.00 | 1.00 |
| 14** | 2 | 2 | 3 | 4 | 1 | 0.40 | 0.13 | 0.20 |
| 15 | 4 | 4 | 4 | 2 | 4 | 0.80 | 0.76 | 0.60 |
| 16 | 3 | 4 | 4 | 3 | 4 | 1.00 | 1.00 | 1.00 |
| 17 | 4 | 3 | 3 | 2 | 4 | 0.80 | 0.76 | 0.60 |
| 18 | 3 | 4 | 4 | 2 | 4 | 0.80 | 0.76 | 0.60 |
| 19 | 3 | 4 | 3 | 2 | 4 | 0.80 | 0.76 | 0.60 |
| 20 | 4 | 4 | 4 | 2 | 4 | 0.80 | 0.76 | 0.60 |
| 21* | 3 | 2 | 4 | 4 | 1 | 0.60 | 0.42 | 0.20 |
| 22 | 3 | 4 | 4 | 2 | 4 | 0.80 | 0.76 | 0.60 |
| 23** | 3 | 2 | 4 | 2 | 1 | 0.40 | 0.13 | -0.20 |
| 24 | 4 | 4 | 4 | 2 | 4 | 0.80 | 0.76 | 0.60 |
| 25 | 4 | 3 | 4 | 2 | 4 | 0.80 | 0.76 | 0.60 |
| 26* | 3 | 2 | 4 | 2 | 4 | 0.60 | 0.42 | 0.20 |
| 27 | 3 | 3 | 4 | 3 | 4 | 1.00 | 1.00 | 1.00 |
| 28* | 3 | 2 | 4 | 4 | 1 | 0.60 | 0.42 | 0.20 |
| 29* | 3 | 2 | 4 | 2 | 4 | 0.60 | 0.42 | 0.20 |
| 30 | 3 | 3 | 3 | 1 | 4 | 0.80 | 0.76 | 0.60 |
| 31 | 3 | 3 | 3 | 3 | 4 | 1.00 | 1.00 | 1.00 |
| 32 | 3 | 4 | 4 | 3 | 4 | 1.00 | 1.00 | 1.00 |
| 33 | 3 | 4 | 4 | 2 | 4 | 0.80 | 0.76 | 0.60 |
| 34 | 3 | 3 | 4 | 3 | 4 | 1.00 | 1.00 | 1.00 |
| 35* | 3 | 2 | 3 | 3 | 1 | 0.60 | 0.42 | 0.40 |
| 36 | 3 | 3 | 2 | 3 | 4 | 0.80 | 0.76 | 0.60 |
| 37** | 4 | 1 | 2 | 3 | 1 | 0.40 | 0.13 | -0.20 |
| 38** | 3 | 1 | 2 | 4 | 1 | 0.40 | 0.13 | -0.20 |

*Items with low I-CVI values (<0.78) but fair kappa score (> 0.40)

**items with low I-CVI values (<0.78) and unacceptable kappa score (<0.4)

Table 1. Items eligibility analysis

| Item | Item-Total Correlation | Skewness | Kurtosis |
| --- | --- | --- | --- |
| 1. I often worry about what my friends are saying about me when I don’t do well in school. | 0.463 | -0.07 | -0.89 |
| 2. I often find myself worrying after I have taken a test even though I know it is too late to do anything about it then. | 0.459 | -0.6 | -0.4 |
| 3. My teachers put a great deal of pressure on me to do well in my classes. | 0.253 | 0.12 | -0.81 |
| 4. It would really upset me if I was doing so poorly in a class at school that my teacher had to notify my parents about it | 0.445 | -0.28 | -1.14 |
| 5. I frequently worry about the restrictions my parents will put on me if I don’t keep my grades up. * | 0.573 | -0.14 | -1.08 |
| 6. It disturbs me when my parents are always after me to spend more time at home on schoolwork. | 0.412 | 0.35 | -0.9 |
| 7. I study hard in all my classes because my parents feel it is very important for me to do well in school. | 0.324 | -0.75 | 0.01 |
| 8. I feel upset when my classmates find out about a low grade I have received in school. * | 0.599 | -0.19 | -0.96 |
| 9. It is very frustrating for me when I cannot seem to learn the things I’m supposed to for school. * | 0.562 | -0.37 | -0.74 |
| 10. I often feel guilty when I do not study when I know I should have. | 0.475 | -0.92 | 0.83 |
| 11.I often worry about what my parents will say when they see the grades I receive on my report card. * | 0.615 | -0.56 | -0.52 |
| 12. I hate the thought of having to tell my parents about a test I haven’t done well on in school. * | 0.571 | 0.16 | -0.94 |
| 13. I get upset when my teachers have to talk to me about not spending enough time on my homework. | 0.469 | 0 | -0.85 |
| 14. It upsets me when I can’t understand the assignments my teacher gives at school. * | 0.594 | -0.36 | -0.75 |
| 15. I usually worry about what my friends and classmates think of me when the teacher calls on me and I don’t know the answer. * | 0.526 | -0.4 | -0.59 |
| 16. I don’t have time to participate in many of the things I would like to because my schoolwork is more important and must come first. | 0.38 | 0.06 | -0.68 |
| 17. It would bother me if my parents were to ask to see a test that I had done poorly on school. * | 0.537 | 0.2 | -0.96 |
| 18. I would worry about what my parents would do to me if they saw a low grade I received at school. * | 0.642 | -0.11 | -0.97 |
| 19. It embarrasses me when the kids at school make fun of me because I can’t answer a question in class. * | 0.586 | -0.04 | -1.09 |
| 20. I always try to do my best in school because it means a lot to get the rewards my parents give me for good grades. | 0.281 | -0.67 | -0.25 |
| 21. It bothers me when my friends ask me about a test I have done poorly on. * | 0.556 | -0.03 | -0.85 |
| 22. I feel a great deal of pressure from my parents to get good grades in school. | 0.471 | 0.03 | -1 |
| 23. It would frustrate me if my parents told me that I should be able to make better grades at school. * | 0.575 | 0.18 | -0.99 |
| 24. It would upset me if my parents made me study more because I didn’t do as well as I should have at school. | 0.435 | 0.17 | -0.77 |
| 25. It would upset me if my teacher had to talk to me about a low grade I had received in school. * | 0.515 | 0.1 | -0.99 |
| 26. It is very important for me to get good grades in all of my schoolwork. | 0.377 | -0.7 | -0.06 |
| 27. It bothers me quite a bit when I don’t do well in school because I’m afraid that my friends and classmates will think I’m stupid. * | 0.524 | 0.05 | -1 |
| 28. I become upset when I begin to study for an important test at school. | 0.295 | 0.74 | 0.01 |
| 29. I often worry about the possibility of not doing well enough in school to get into college. * | 0.572 | -0.6 | -0.27 |
| 30. I worry about the possibility of disappointing my parents if I don’t do well in school. * | 0.600 | -0.87 | 0.37 |
| 31. I would worry about what my parents would do to me if my teacher had to notify them about my work at school. * | 0.593 | -0.14 | -0.82 |
| 32. It would disturb me if my teacher said I was not trying in class because I didn’t do as well as the school thought I should do. * | 0.530 | 0 | -0.87 |
| 33. My schoolwork must always come first because my teachers feel it is important for me to study and learn. | 0.331 | -0.65 | 0.17 |
| 34. I am worried that I may leave school without any qualifications. | 0.429 | -0.23 | -1.01 |

*Note.* * Item total correlation (*r* *_it_* >0.50), skewness (<|2|) and kurtosis (<|4|); included in EFA.

Table 3. SIVASS Content Validation Results

| Item | Expert 1 | Expert 2 | Expert 3 | Expert4 | Expert 5 | I-CVI | Kappa | CVR |
| --- | --- | --- | --- | --- | --- | --- | --- | --- |
| 8 | 3 | 4 | 4 | 4 | 4 | 0.80 | 0.76 | 0.60 |
| 9 | 2 | 4 | 3 | 3 | 4 | 0.80 | 0.76 | 0.60 |
| 11 | 3 | 4 | 4 | 3 | 4 | 1.00 | 1.00 | 1.00 |
| 12 | 3 | 3 | 4 | 3 | 4 | 1.00 | 1.00 | 1.00 |
| 14 | 3 | 4 | 4 | 3 | 4 | 1.00 | 1.00 | 1.00 |
| 17 | 3 | 4 | 4 | 3 | 4 | 1.00 | 1.00 | 1.00 |
| 18 | 3 | 4 | 3 | 2 | 4 | 0.80 | 0.76 | 0.60 |
| 19 | 4 | 4 | 4 | 2 | 4 | 0.80 | 0.76 | 0.60 |
| 21 | 3 | 4 | 4 | 2 | 4 | 0.80 | 0.76 | 0.60 |
| 23 | 4 | 3 | 4 | 2 | 4 | 0.80 | 0.76 | 0.60 |
| 25 | 3 | 3 | 4 | 3 | 4 | 1.00 | 1.00 | 1.00 |
| 27 | 3 | 2 | 4 | 3 | 4 | 0.80 | 0.76 | 0.60 |
| 29 | 3 | 3 | 3 | 3 | 4 | 1.00 | 1.00 | 1.00 |
| 30 | 3 | 4 | 4 | 3 | 4 | 1.00 | 1.00 | 1.00 |
| 31 | 3 | 4 | 4 | 2 | 4 | 0.80 | 0.76 | 0.60 |
| 32 | 3 | 3 | 4 | 3 | 4 | 1.00 | 1.00 | 1.00 |

1. Scree plot of SIVASS exploratory factor analysis


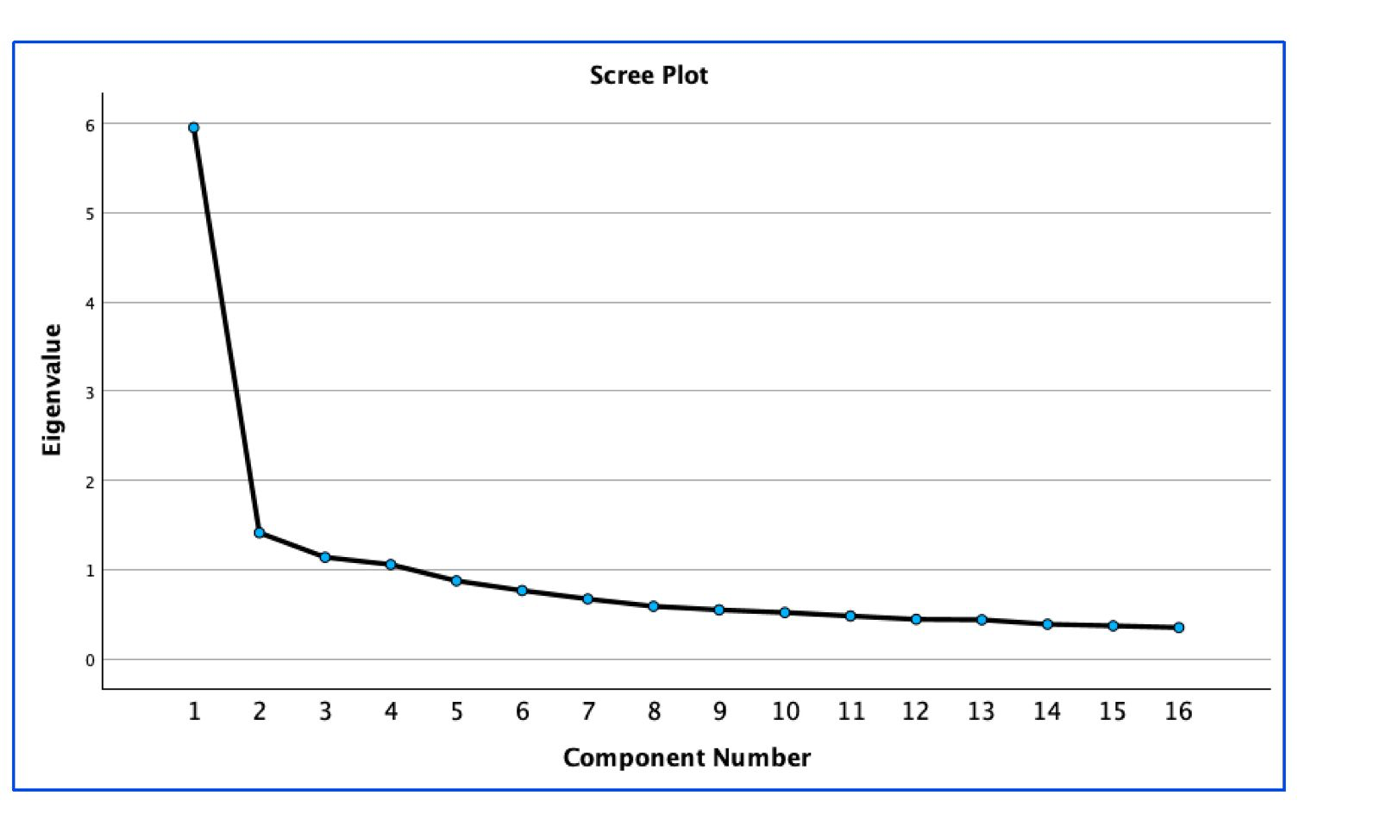


Figure 1. Scree plot of SIVASS exploratory factor analysis

1. SIVASS confirmatory factor analysis results

Table 4. SIVASS confirmatory factor analysis results

| **Item** | Factors | | | | | | | |
| --- | --- | --- | --- | --- | --- | --- | --- | --- |
|  | Parent-related  pressure | | Internal-related  pressure | | Friend-related  pressure | | Teacher-related  pressure | |
|  | **Estimate** | **Std. All** | **Estimate** | **Std. All** | **Estimate** | **Std. All** | **Estimate** | **Std. All** |
| 12 | 1.000 | 0.669 |  |  |  |  |  |  |
| 18 | 1.044 | 0.683 |  |  |  |  |  |  |
| 17 | 1.164 | 0.759 |  |  |  |  |  |  |
| 23 | 0.938 | 0.619 |  |  |  |  |  |  |
| 31 | 0.967 | 0.688 |  |  |  |  |  |  |
| 11 | 0.885 | 0.644 |  |  |  |  |  |  |
| 9 |  |  | 1.000 | 0.662 |  |  |  |  |
| 29 |  |  | 0.995 | 0.717 |  |  |  |  |
| 14 |  |  | 0.940 | 0.646 |  |  |  |  |
| 30 |  |  | 0.939 | 0.729 |  |  |  |  |
| 27 |  |  |  |  | 1.000 | 0.645 |  |  |
| 19 |  |  |  |  | 1.323 | 0.813 |  |  |
| 21 |  | 1.048 |  |  | 1.052 | 0.737 |  |  |
| 8 |  |  |  |  | 1.097 | 0.743 |  |  |
| 32 |  |  |  |  |  |  | 1.000 | 0.758 |
| 25 |  |  |  |  |  |  | 0.974 | 0.682 |
| **Second order** | 1.000 | 0.862 | 0.968 | 0.849 | 1.048 | 0.883 | 1.020 | 0.812 |

4. SIVASS questionnaire

|  | English back translation | SIVASS | Sangat Tidak setuju  *Strongly disagree* | Tidak Setuju  *disagree* | Tidak dapat menentukan  *Undecided* | Setuju  *agree* | Sangat setuju  *Strongly agree* |
| --- | --- | --- | --- | --- | --- | --- | --- |
| 8 | I feel upset when my classmates find out about a low grade I have received in school. | Saya merasa kesal/ kecewa ketika teman sekelas mengetahui bahwa saya memperoleh nilai rendah di sekolah |  |  |  |  |  |
| 9 | It is very frustrating for me when I can’t seem to learn the things I’m supposed to for school. | Saya menjadi sangat frustasi ketika saya sepertinya kurang mampu mempelajari sesuatu yang seharusnya saya dapat pelajari di sekolah. |  |  |  |  |  |
| 11 | I often worry about what my parents will say when they see the grades I receive on my report card. | Saya sering khawatir mengenai apa yang akan orang tua saya katakan ketika mereka melihat nilai rapor saya. |  |  |  |  |  |
| 12 | I hate the thought of having to tell my parents about a test I haven’t done well on in school. | Saya membenci pikiran bahwa saya perlu memberitahu orang tua mengenai ulangan atau tes yang kurang dapat saya kerjakan dengan baik di sekolah. |  |  |  |  |  |
| 14 | It upsets me when I can’t understand the assignments my teacher gives at school. | Saya menjadi kesal/kecewa ketika saya tidak dapat memahami tugas yang diberikan guru di sekolah. |  |  |  |  |  |
| 17 | It would bother me if my parents were to ask to see a test that I had done poorly on. | Saya akan menjadi terganggu jika orang tua saya ingin melihat ulangan yang saya kerjakan dengan buruk. |  |  |  |  |  |
| 18 | I would worry about what my parents would do to me if they saw a low grade I received at school. | Saya akan menjadi khawatir mengenai apa yang orang tua akan lakukan terhadap saya jika melihat nilai rendah yang saya dapatkan di sekolah. |  |  |  |  |  |
| 19 | It embarrasses me when the kids at school make fun of me because I can’t answer a question in class. | Saya menjadi malu jika teman-teman di sekolah mengolok-olok karena saya tidak mampu menjawab pertanyaan di kelas. |  |  |  |  |  |
| 21 | It bothers me when my friends ask me about a test I have done poorly on. | Saya menjadi terganggu ketika teman-teman saya menanyakan tentang ulangan yang saya kerjakan dengan buruk |  |  |  |  |  |
| 23 | It would frustrate me if my parents told me that I should be able to make better grades at school. | Saya akan menjadi frustasi jika orang tua saya mengatakan bahwa saya seharusnya bisa mendapatkan nilai yang lebih baik di sekolah. |  |  |  |  |  |
| 25 | It would upset me if my teacher had to talk to me about a low grade I had received in school. | Saya akan menjadi kesal/kecewa jika guru perlu berbicara kepada saya mengenai nilai rendah yang saya terima di sekolah. |  |  |  |  |  |
| 27 | It bothers me quite a bit when I don’t do well in school because I’m afraid that my friends and classmates will think I’m stupid. | Saya menjadi cukup terganggu ketika saya tidak tidak mengerjakan sesuatu dengan baik di sekolah, karena saya takut bahwa teman-teman dan teman sekelas saya akan berpikir bahwa saya bodoh. |  |  |  |  |  |
| 29 | I often worry about the possibility of not doing well enough in school to get into college. | Saya sering khawatir bahwa ada kemungkinan saya tidak melakukan yang terbaik di sekolah untuk dapat diterima di perguruan tinggi. |  |  |  |  |  |
| 30 | I worry about the possibility of disappointing my parents if I don’t do well in school. | Saya khawatir bahwa ada kemungkinan saya mengecewakan orang tua jika tidak berprestasi baik di sekolah. |  |  |  |  |  |
| 31 | I would worry about what my parents would do to me if my teacher had to notify them about my work at school. | Saya akan menjadi khawatir tentang apa yang akan orang tua lakukan kepada saya jika guru perlu memberitahu mereka mengenai hasil kerja saya di sekolah. |  |  |  |  |  |
| 32 | It would disturb me if my teacher said I was not trying in class because I didn’t do as well as the school thought I should do. | Saya akan menjadi terganggu jika guru mengatakan bahwa saya tidak berusaha di dalam kelas karena tidak melakukan apa yang sebaiknya dilakukan seperti yang diharuskan oleh sekolah |  |  |  |  |  |
